# Loss of NLRP3 reduces oxidative stress and polarizes intratumor macrophages to attenuate immune attack on endometrial cancer

**DOI:** 10.3389/fimmu.2023.1165602

**Published:** 2023-04-03

**Authors:** Xiaolu Zhu, Yanli Xu, Juan Wang, Zhuowei Xue, Tian Qiu, Jing Chen

**Affiliations:** Department of Obstetrics and Gynecology, Shanghai Sixth People’s Hospital Affiliated to Shanghai Jiao Tong University School of Medicine, Shanghai, China

**Keywords:** endometrial cancer (EMC), tumor-associated macrophages (TAMs), reactive oxygen species (ROS), NOD-like receptor family, pyrin domain-containing 3 (NLRP3), macrophage polarization, inflammation

## Abstract

**Introduction:**

The interaction between endometrial cancer (EMC) cells and intratumoral macrophages plays a significant role in the development of the disease. PYD domains-containing protein 3 (NLRP3) inflammasome formation triggers caspase-1/IL-1β signaling pathways and produces reactive oxygen species (ROS) in macrophages. However, the role of NLRP3-regulated ROS production in macrophage polarization and the subsequent growth and metastasis of EMC remains unknown.

**Methods:**

We conducted bioinformatic analysis to compare NLRP3 levels in intratumoral macrophages from EMC and normal endometrium. *In vitro* experiments involved knocking out NLRP3 in macrophages to shift the polarization from an anti-inflammatory M1-like phenotype to a proinflammatory M2-like phenotype and reduce ROS production. The impact of NLRP3 depletion on the growth, invasion, and metastasis of co-cultured EMC cells was assessed. We also evaluated the effect of NLRP3 depletion in macrophages on the growth and metastasis of implanted EMC cells in mice.

**Results:**

Our bioinformatic analysis showed significantly lower NLRP3 levels in intratumoral macrophages from EMC than those from normal endometrium. Knocking out NLRP3 in macrophages shifted their polarization to a proinflammatory M2-like phenotype and significantly reduced ROS production. NLRP3 depletion in M2-polarized macrophages increased the growth, invasion, and metastasis of co-cultured EMC cells. NLRP3 depletion in M1-polarized macrophages reduced phagocytic potential, which resulted in weakened immune defense against EMC. Additionally, NLRP3 depletion in macrophages significantly increased the growth and metastasis of implanted EMC cells in mice, likely due to compromised phagocytosis by macrophages and a reduction in cytotoxic CD8+ T cells.

**Discussion:**

Our results suggest that NLRP3 plays a significant role in regulating macrophage polarization, oxidative stress, and immune response against EMC. NLRP3 depletion alters the polarization of intratumoral macrophages, leading to weakened immune defense against EMC cells. The reduction in ROS production by the loss of NLRP3 may have implications for the development of novel treatment strategies for EMC.

## Introduction

Endometrial cancer (EMC) is a common form of epithelial cancer that affects the endometrium in women approaching or past menopause (1). It is one of the most prevalent tumors in the female reproductive system, with approximately 200,000 new cases reported annually, and is the third deadliest gynecological cancer after cervical and ovarian cancer (2). In North America and Europe, endometrial cancer ranks as the fourth most common type of cancer after breast, lung, and colorectal cancer and is the most diagnosed cancer in the female reproductive system (3). EMC occurrence can be affected by lifestyle, since obesity, some metabolic syndromes, and tamoxifen and estrogen treatment all increase the risk of EMC (4).

Recent studies have shed light on the importance of the cancer microenvironment to the tumorigenesis of EMC. Intratumor macrophages or tumor-associated macrophages (TAMs) play critical roles in the initiation, progression, invasion, and migration of EMC cells (5, 6). The regulation in the EMC microenvironment involves the differentiation and polarization of TAMs in response to the signaling of EMC cells and the morphologic alteration and molecular changes in EMC cells in response to the cytokines, chemokines, and trophic factors produced and secreted by TAMs (7, 8). Macrophage polarization refers to the display of a macrophage’s phenotype as either a pro-inflammatory type, labeled “M1,” or an anti-inflammatory type, designated “M2” (9). Newly created macrophages are referred to as “naive macrophages” or “M0” (10). TAMs are a heterogeneous group of intratumoral macrophages that exhibit varying levels of polarization. Macrophages closer to an M2-like phenotype tend to be anti-tumoral, whereas those more like an M1-like phenotype tend to be pro-tumoral. This highlights the dynamic nature of TAMs and their ability to influence the tumor microenvironment in different ways, making them an important area of study in cancer biology. Understanding the relationship between TAM polarization and tumor behavior could lead to the development of new and more effective cancer therapies. *In vitro* polarization of bone marrow-derived macrophages (BMDMs) is a widely used method for study. BMDMs are first obtained from bone marrow as naive (M0) macrophages (11). To induce M1 polarization, M0 macrophages are treated with 20 ng/ml macrophage colony-stimulating factor (M-CSF) and 30 ng/ml lipopolysaccharides (LPS). To induce M2 polarization, M0 macrophages are treated with 20 ng/ml IL-4 and 20 ng/ml IL-13 (12). This approach allows researchers to study the different functions and behaviors of M1 and M2 macrophages and provides insight into the role of macrophage polarization in various biological processes.

The molecular regulation of the polarization of TAMs in EMC is not fully understood. Interleukin-1β (IL-1β) is a cytokine that plays a key role in regulating both the inflammatory response to tumors and the immune response to tumor cells. IL-1β is produced by a variety of immune cells, including macrophages, neutrophils, and lymphocytes, as well as antigen-presenting cells. By influencing the behavior of these cells, IL-1β helps to shape the overall immune response to the tumor, making it an important player in the complex interplay between the immune system and cancer (13). Inflammasomes, like the NOD-like receptor family, pyrin domain-containing 3 (NLRP3), are critical regulators for IL-1β activation through catalyzing the conversion of pro-IL-1β into its active form (14). The NLRP3 inflammasome can be activated by oxidative stress and, in turn, increase the production of reactive oxygen species (ROS), particularly in macrophages (15). ROS are highly reactive molecules that can cause oxidative stress, leading to significant damage to cellular components (16). Additionally, ROS can activate signaling pathways that regulate cellular processes, such as cell cycle progression, survival, and migration (16). The accumulation of ROS within cells can have a range of detrimental effects, including cellular damage, cellular dysfunction, and even cell death (16). However, under normal physiological conditions, ROS levels are tightly regulated and serve as important signaling molecules that play a role in various cellular processes (16). Hence, regulation of ROS production by the NLRP3 inflammasome can be either anti-tumorigenic by triggering cellular stress responses and apoptosis or pro-tumorigenic by promoting angiogenesis, inflammation, and immune suppression (17, 18). Thus, the regulation of ROS production by the NLRP3 inflammasome is complex and context-dependent, and further research is needed to fully understand the role of the NLRP3 inflammasome in intratumor macrophages in the regulation of ROS production and macrophage polarization as well as its impact on tumorigenesis.

## Materials and methods

### Ethical approval

All experiments, including animal work, were conducted in accordance with the guidelines set by the Institutional Animal Research Ethics Council of Shanghai Jiao Tong University School of Medicine. Approval was obtained for these experiments prior to their initiation. It should be noted that this study did not involve the use of human specimens.

### Animals

The role of macrophage-derived NLRP3 in EMC was studied by transplanting a human EMC cell line (HEC-1A) subcutaneously into mice with macrophage depletion of NLRP3 [Lysosome2 (Lys2)-Cre; NLRP3 (fx/fx)] or control NLRP3 (fx/fx) mice. A total of 10^7^ EMC cells were used in the transplantation procedure, and the formation of tumors and metastasis was evaluated over a period of 90 days. The HEC-1A cell line was obtained from the American Type Culture Collection (ATCC, HTB-112, Rockville, MD, USA). The Lys2-Cre and NLRP3 (fx/fx) strains were obtained from Jax mice (Strain Nos. 018956 and 017970).

### EMC cell line and modulation of -rimary macrophages

A human EMC cell line (HEC-1A, a subclone of HEC-1B (HTB-113, ATCC)) that was isolated from endometrial adenocarcinoma of a 71-year-old patient (19). The HEC-1A cells were grown in Dulbecco’s Modified Eagle’s Medium (DMEM) containing 7.5% fetal bovine serum (FBS, sourced from Invitrogen in Carlsbad, CA, USA) in a 37°C incubator with 5% CO_2_ atmospheric conditions to maintain a moist environment. To allow *in vivo* tracing of grafted HEC-1A cells, they were transduced with lentiviral luciferase (LVR-1048, Cellomics Technology, LLC, Halethorpe, MD, USA). The tumor mass was evaluated based on bioluminescence with a luciferin assay.

Bone marrow was obtained from mice and transferred to six-well plates filled with 3 ml/well DMEM at a density of 2.5 x 106 cells/ml. Only attached cells were maintained (naive macrophages; M0) by washing them every 3 h with DMEM containing 8% FBS and 20 ng/ml M-CSF for 8 days. M0 macrophages were induced to become M1 by 20 ng/ml M-CSF and 30 ng/ml LPS or to become M2 by 20 ng/ml IL-4 and 20 ng/ml IL-13.

### ELISA

The total protein was extracted from both the cultured cells and the isolated cells from mouse tissue and then subjected to an enzyme-linked immunosorbent assay (ELISA) using specific kits designed for mouse NLRP3 (ab279417; Abcam, Cambridge, MA, USA), mouse IL-1β (ab197742; Abcam), mouse tumor necrosis factor alpha (TNFα, ab208348; Abcam), mouse interferon gamma (IFNγ, ab282874; Abcam), mouse CD163 (ab272204; Abcam), and mouse arginase 1 (ARG1, ab269541; Abcam).

### Cell proliferation, invasion, and migration assay

Cell proliferation was measured by counting the number of live cells using the Cell Counting Kit-8 (CCK-8) assay (CCK-8, Sigma-Aldrich). The Transwell cell invasion assay was used to measure the ability of cancer cells to invade through a porous membrane into the underlying matrix, for which cancer cells were placed on top of a porous membrane in a Transwell insert while serum-free media was added in the lower chamber. After 24 h, the migrated cells were fixed, stained, and counted. The Transwell cell migration assay was used to measure the ability of cells to migrate through a porous membrane into the lower chamber filled with serum-free media. After 24 h, the migrated cells were fixed, stained, and counted.

### Flow cytometry

For flow cytometry analysis, the tumor was carefully dissected, after which single cells were obtained through a digestion process using 0.25% trypsin (Invitrogen) for 45 min. These cells were then labeled with PE-cy7-conjugated CD3 and BV421-conjugated CD8 antibodies (Becton-Dickinson Biosciences, Shanghai, China). The flow cytometry results were displayed using FlowJo software (FlowJo LLC, Ashland, OR, USA).

### ROS assay

A cellular reactive oxygen species detection assay kit (ab186027, Abcam) used the dichlorodihydrofluorescein diacetate (DCF-DA) method to measure the levels of ROS in macrophages. Briefly, DCF-DA was added to cultured cells and then taken up and converted into the fluorescent molecule dichlorodihydrofluorescein (DCF) by intracellular esterases. ROS in the cells oxidized DCF, resulting in an increase in fluorescence. The relative fluorescence (RFU) was then measured using a fluorimeter, serving as an indirect quantification of cellular ROS.

### Analysis of phagocytosis

Phagocytosis, the process by which cells engulf and internalize foreign particles, was evaluated using two different methods. The first method involved using a zymosan-based phagocytic kit (ab211156, Abcam) to assess the uptake of zymosan particles by macrophages after a 30-minute incubation period. The second method used to assess phagocytosis involved counting the number of green fluorescent protein (GFP)-labeled bacteria that were internalized by the cells by flow cytometry (20). The GFP-labeled bacteria were prepared by transforming bacteria with a GFP-containing plasmid through electroporation. The GFP-labeled bacteria were used to visualize the process of phagocytosis.

### Statistics and bioinformatics

The data collected from the experiments was analyzed using the statistical software GraphPad Prism 7 (GraphPad, Chicago, IL, USA). The analysis was performed using an unpaired student T-test to compare the individual values, and the mean and standard deviation (SD) were calculated. The results were considered statistically significant if the p-value was less than 0.05. In addition to statistical analysis, bioinformatics techniques were also used here. A GEO database (GSE117970) was utilized to compare the gene expression between macrophages isolated from EMC and those isolated from normal endometrium (NEM). The R software was used to identify differentially expressed genes, while the Metascape (https://metascape.org/gp/index.html#/main/step1) and String (https://string-db.org/) online tools were employed to analyze the pathway expression and gene network. These bioinformatics tools provide a comprehensive understanding of the genetic information and the biological pathways involved in the process under investigation.

## Results

### NLRP3 levels are significantly reduced in macrophages from EMC compared to NEM

**Figure 1:**
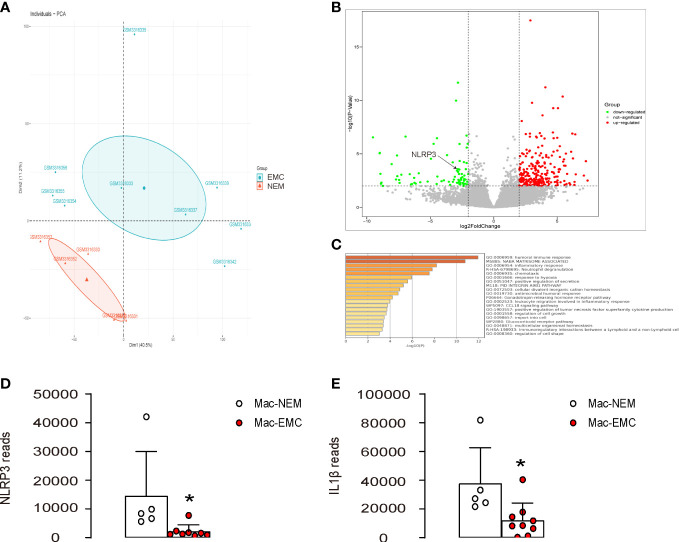
NLRP3 levels are significantly reduced in macrophages from EMC compared to NEM. The expression of NLRP3 was examined in macrophages from EMC and NEM, using a GEO database GSE117970. **(A)** PCA plot. **(B)** A volcano map to show significant downregulation of NLRP3 in EMC-macrophages. **(C)** Pathway analysis of differentially expressed genes by Metascape. **(D, E)** Array reads for NLRP3 **(D)** and IL-1β **(E)** in macrophages from EMC and NEM. *p <0.05.

To investigate the role of NLRP3 in macrophages in the interaction between macrophages and EMC, we analyzed the expression of NLRP3 in macrophages from EMC and NEM using a GEO database (GSE117970). The results showed a distinct pattern of gene expression in EMC-macrophages compared to NEM-macrophages, as revealed by the principal component analysis (PCA) plot (**Figure 1A**). Further analysis identified NLRP3 as a differentially expressed gene that was significantly downregulated in EMC-macrophages (**Figure 1B**). Additionally, the expression of many signaling pathways related to macrophage differentiation, polarization, and immune responses was altered in EMC-macrophages compared to NEM-macrophages (**Figure 1C**). Interestingly, not only was the expression of NLRP3 significantly decreased in EMC-macrophages compared to NEM-macrophages (**Figure 1D**), but also the expression of IL-1β, a downstream effector of NLRP3, was significantly reduced in EMC-macrophages compared to NEM-macrophages (**Figure 1E**). These findings suggest that NLRP3 levels are significantly reduced in macrophages from EMC compared to NEM.

### NLRP3 depletion in macrophages reduces ROS production and M2-like macrophage polarization

**Figure 2:**
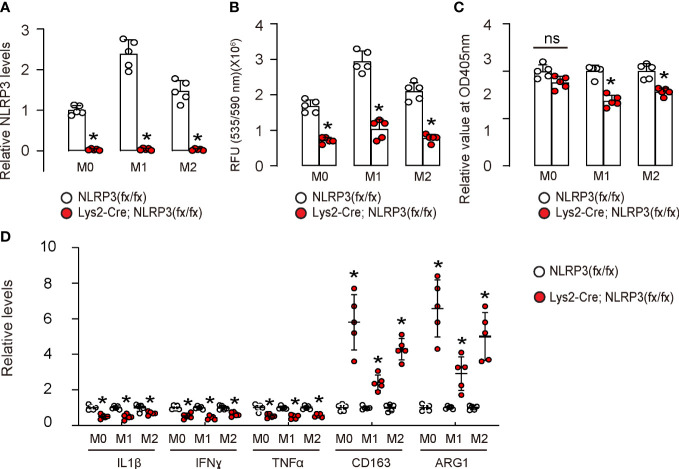
NLRP3 depletion in macrophages reduces ROS production and M2-like macrophage polarization. We generated mice with macrophage-specific depletion of NLRP3, Lys2-Cre; NLRP3(fx/fx) mice and control NLRP3(fx/fx) mice. BMDMs were isolated from Lys2-Cre; NLRP3(fx/fx) mice as M0, which were further differentiated into M1 macrophages by treatment with IPS and IFNγ or differentiated into M2 macrophages by treatment with IL-4 and IL-13. **(A)** The NLRP3 depletion was confirmed in all M0, M1, and M2 macrophages from Lys2-Cre; NLRP3(fx/fx) mice by ELISA. **(B)** Measurement of ROS production in all NLRP3-depleted or control M0, M1, and M2 macrophages by ROS assay. **(C)** CCK-8 assay for 72 h to measure the number of total macrophages. **(D)** ELISA for IL-1β, IFNγ, TNFα, CD163, and arginase 1 (ARG1) in macrophages. ns, non-significant. *p <0.05. N = 5.

Next, we assessed the effects of NLRP3 depletion on macrophages. First, we generated mice with macrophage-specific depletion of NLRP3, Lys2-Cre; NLRP3(fx/fx), and control NLRP3(fx/fx). BMDMs were extracted from Lys2-Cre; NLRP3(fx/fx) mice, untreated as M0 phenotypes. These M0 macrophages were further differentiated into either M1 or M2 macrophages, with M1 macrophages being induced through treatment with IPS and IFNγ and M2 macrophages being generated with treatment with IL-4 and IL-13. The NLRP3 depletion was confirmed in all M0, M1, and M2 macrophages from the Lys2-Cre; NLRP3(fx/fx) mice by ELISA (**Figure 2A**). Moreover, NLRP3 depletion significantly reduced ROS production in all M0, M1, and M2 macrophages on the ROS assay (**Figure 2B**). Furthermore, NLRP3 depletion significantly reduced the total number of M1 or M2 macrophages in 72-h culture but did not alter the total number of M0 macrophages (**Figure 2C**), suggesting that NLRP3 may play a role in the growth of differentiated macrophages rather than naïve macrophages. The M1/M2 markers were examined in these macrophages by ELISA, showing that NLRP3 depletion significantly reduced the levels of M1 markers/proinflammatory factors IL-1β, IFNγ, and TNFα and significantly increased the levels of M2 markers/anti-inflammatory factors CD163 and arginase 1 (ARG1) (**Figure 2D**). These data suggest that NLRP3 depletion in macrophages reduces ROS production and M2-like macrophage polarization, which could contribute to tumorigenesis and progression. The alteration in these M1/M2 markers was likely more pronounced in M0 macrophages than M1 or M2 macrophages, which may be due to the relative ease with which naïve macrophages can be polarized by NLRP3 modification compared to the repolarization of differentiated macrophages.

### NLRP3-depletion in M2-polarized macrophages increases growth, invasion, and migration potential of co-cultured EMC cells

**Figure 3:**
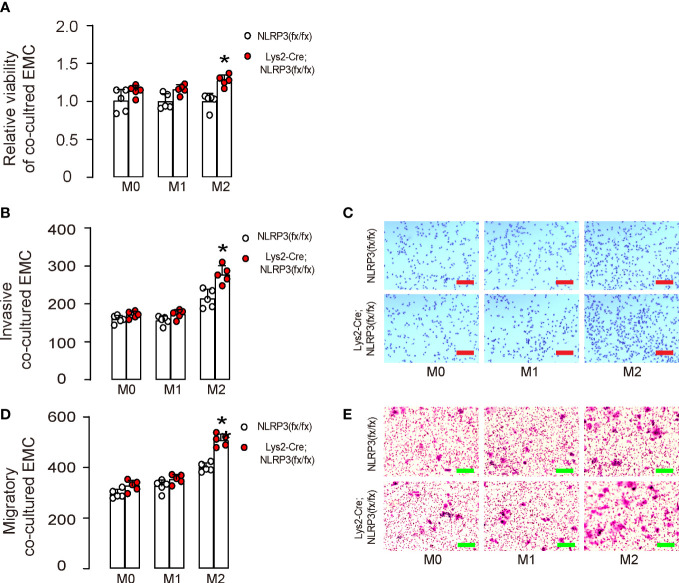
NLRP3-depletion in macrophages increases the growth, invasion, and migration potential of co-cultured EMC cells. BMDMs were isolated from Lys2-Cre; NLRP3(fx/fx) mice as M0, which were further differentiated into M1 macrophages by treatment with IPS and IFNγ or differentiated into M2 macrophages by treatment with IL-4 and IL-13. **(A–E)** M0, M1, or M2 macrophages were co-cultured with HEC-1A. **(A)** CCK-8 assay for growth of HEC-1A cells. **(B, C)** Invasion of HEC-1A cells, shown by quantification **(B)** and by representative images **(C)**. **(D, E)** Migration potential of HEC-1A cells, shown by quantification **(D)** and by representative images **(E)**. *p <0.05. N = 5. Scale bars are 100 µm.

Next, M0, M1, or M2 macrophages were co-cultured with HEC-1A. We found that NLRP3-depletion in M2-polarized macrophages, but not in M0- or M1-polarized macrophages, significantly increased the growth of co-cultured HEC-1A cells (**Figure 3A**). Moreover, NLRP3-depletion in M2-polarized macrophages but not in M0- or M1-polarized macrophages significantly increased the invasion of co-cultured HEC-1A cells (**Figures 3B, C**). Furthermore, NLRP3-depletion in M2-polarized macrophages but not in M0- or M1-polarized macrophages significantly increased the migration potential of co-cultured HEC-1A cells (**Figures 3D, E**). Thus, our data suggest that NLRP3-depletion in M2-polarized macrophages increases the growth, invasion, and migration potential of co-cultured EMC cells. We discovered that the depletion of NLRP3 in M2-polarized macrophages, but not in M0- or M1-polarized macrophages, resulted in a significant increase in the growth of co-cultured HEC-1A cells (**Figure 3A**). Moreover, our findings showed that NLRP3-depletion in M2-polarized macrophages also significantly enhanced the invasiveness (**Figures 3B, C**) and the migratory capacity (**Figures 3D, E**) of the co-cultured HEC-1A cells. Therefore, our study suggests that the depletion of NLRP3 in M2-polarized macrophages leads to a significant increase in the growth, invasiveness, and migratory capacity of the co-cultured EMC cells.

### NLRP3 depletion in M1-polarized macrophages results in reduced phagocytosis

**Figure 4:**
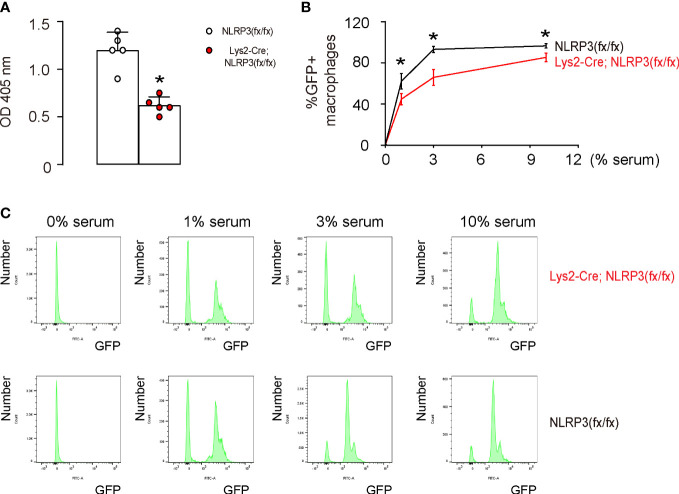
NLRP3 depletion in M1-polarized macrophages results in reduced phagocytosis. **(A)** A phagocytosis assay that analyzes 30 min zymosan intake. **(B, C)** A flow cytometry assay to analyze the intake of GFP+ bacteria, shown by the percentage of the GFP+ macrophages **(B)** and by representative flow charts **(C)**. *p <0.05. N = 5.

The function of phagocytosis is crucial to the anti-tumor properties of M1 macrophages. To examine the impact of NLRP3 depletion in M1-polarized macrophages on phagocytosis, we conducted two different assessments. First, we used a phagocytosis assay that analyzed the zymosan intake over a period of 30 min and observed a significant reduction in the phagocytosis capability of M1-polarized macrophages from Lys2-Cre and NLRP3(fx/fx) mice (**Figure 4A**). Additionally, using flow cytometry to measure the intake of GFP+ bacteria by M1-polarized macrophages, we found that there was a significant decrease in the percentage of GFP+ macrophages, as shown by quantification (**Figure 4B**) and by representative flow charts (**Figure 4C**). This evidence suggests that NLRP3 depletion in M1-polarized macrophages results in a decrease in phagocytic activity.

### NLRP3 depletion promotes growth and metastasis of EMC *in vivo*

**Figure 5:**
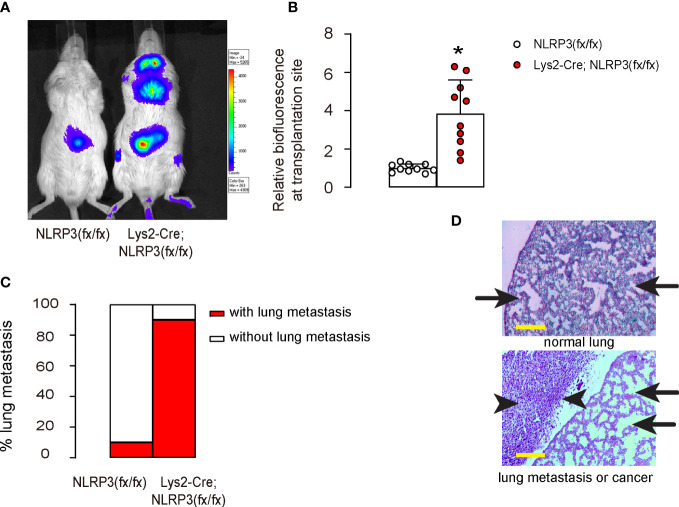
NLRP3 depletion promotes growth and metastasis of EMC *in vivo*. Luciferase-transduced HEC-1A cells were subcutaneously transplanted into mice with macrophage-depletion of NLRP3, Lys2-Cre; NLRP3(fx/fx) and control NLRP3(fx/fx). The tumor formation and metastasis were tested after 90 days. **(A, B)** Bioluminescence in a luciferin assay, shown by representative images **(A)** and by quantification **(B)**. **(C)** Incidence of lung metastasis. **(D)** Flow cytometry analysis for cytotoxic CD3+CD8+ T cells in tumors. Arrows pointed to lung tissue, and arrowheads pointed to a metastatic tumor in the lung. *p <0.05. N = 10 in each group. Scale bars are 100 µm.

The effect of macrophage-mediated depletion of NLRP3 on tumor growth and metastasis was tested *in vivo*. Luciferase-transduced HEC-1A cells were subcutaneously transplanted into mice with macrophage-depletion of NLRP3, Lys2-Cre; NLRP3(fx/fx), and control NLRP3(fx/fx). The tumor formation and metastasis were tested after 90 days. We found that NLRP3 depletion in macrophages significantly increased the growth (**Figures 5A, B**) and metastasis (**Figures 5C, D**) of the implanted EMC cells in mice.

### NLRP3-depletion induced M2 macrophage polarization reduces cytotoxic T cells *in vivo*

**Figure 6:**
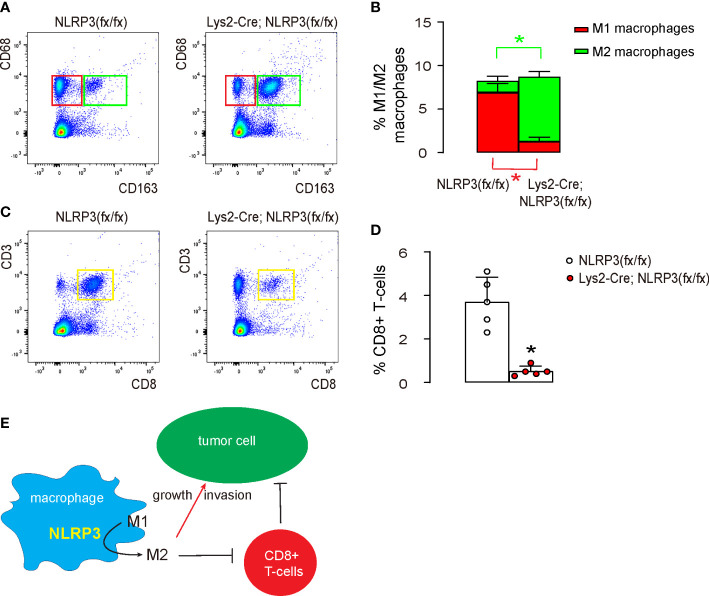
NLRP3-depletion-induced M2 macrophage polarization reduces cytotoxic T cells *in vivo*. The tumor was digested and subjected to flow cytometry analysis. **(A, B)** FACS for CD68 and CD163, shown by quantification **(A)** and by representative flow charts **(B)**. **(C, D)** FACS for CD3 and CD8, shown by quantification **(C)** and by representative flow charts **(D)**. **(E)** Schematic of the study, showing that loss of NLRP3 polarizes intratumor macrophages to attenuate the immune attack on EMC both directly and indirectly through regulating cytotoxic T cells. *p <0.05. N = 5.

Finally, we investigated the underlying mechanisms. We found that tumor implantation into macrophagic NLRP3-depleted mice significantly reduced CD163-CD68+ M1 macrophages and significantly increased CD163+CD68+ M2 macrophages compared to tumor implantation into control mice (**Figures 6A, B**). Moreover, the shift in macrophage polarization in macrophagic NLRP3-depleted mice resulted in a significant reduction in CD3+CD8+ cytotoxic T cells, the main cancer killer cells (**Figures 6C, D**). Together, our data suggest that reduced oxidative stress caused by the loss of NLRP3 polarizes intratumor macrophages to attenuate the immune attack on EMC (**Figure 6E**).

## Discussion

Crosstalk between TAMs and EMC cells orchestrates the tumorigenesis, growth, invasion, and metastasis of EMC (5, 6). However, the molecular signaling underlying this interactive regulation is known to be a complicated and dynamic process but is not well described (21). Importantly, the alteration in the altered genes associated with macrophage polarization resulted in reduced phagocytosis of M1 macrophages and enhanced supportive effects of M2 macrophages on tumor cell growth and metastasis (10). Since both effects are detrimental to the control of EMC, our data suggest that increasing expression of NLRP3 in macrophages may help to suppress the growth and metastasis of EMC.

Controlled by sex steroids, IL-1α is known to play a pivotal role in the carcinogenesis of EMC (22). Here, the reduction in NLRP3 and IL-1β in EMC indicates a possible interaction between IL-1α and IL-1β in EMC since they share the same receptor (IL-1R1) on the endometrium (22). Moreover, as the affinity for IL-1R1 of IL-1α and IL-1β is different, their competition for binding IL-1R1 may result in a change in downstream signaling, which affects the tumorigenesis of EMC (23).

The oxidation of tumor cells, also known as oxidative stress, can have a significant impact on tumor growth and invasion (24), and was found here to be regulated by NLRP3 in intratumoral macrophages. Additionally, oxidative stress can also activate signaling pathways that regulate cell proliferation, survival, and migration (25), all of which are key processes in tumor-associated angiogenesis, tumor growth and invasion. Since here we found that NLRP3-controlled oxidative stress regulated macrophage polarization, it suggests that NLRP3 expression in TAMs may affect the behavior of immune cells to regulate the tumor microenvironment, promoting inflammation and immune suppression, both of which can contribute to tumor growth, and invasion (26). On the other hand, it is also worth mentioning that oxidative stress can trigger cellular stress responses and apoptosis, leading to the death of damaged or mutated cells and inhibiting tumor growth and invasion (26). Thus, the impact of oxidative stress on tumor growth and invasion is complex and can have both promoting and inhibiting effects, depending on the specific context and type of tumor. ROS and MPO (myeloperoxidase) are two biologically important molecules that are related in the context of inflammation and oxidative stress (27). ROS are highly reactive, short-lived molecules that are produced because of cellular metabolism and exposure to environmental stressors (27). MPO is an enzyme that is expressed and produced by immune cells such as neutrophils and monocytes and is involved in the generation of ROS (27). In the context of inflammation and oxidative stress, MPO can convert hydrogen peroxide (H_2_O_2_) into highly reactive and toxic molecules such as hypochlorite (ClO^−^) and chloramines, which contribute to tissue damage and oxidative stress (27). This process can lead to the production of additional ROS, amplifying the oxidative stress response (27). Since we found that ROS expression is reduced in NLRP3-depleted macrophages, it may be interesting to examine the changes in MPO in a future study.

The Toll-like receptor 4 (TLR4) and nuclear factor kappa B (NF-kB) pathways play a crucial role in the innate immune response to bacterial LPS (28). This pathway is activated when TLR4 recognizes LPS and triggers NF-kB, which then regulates the expression of pro-inflammatory cytokines and other genes involved in the immune response, likely through the NLRP3 protein (28). In the absence of NLRP3, the activation of the TLR4/NF-kB pathway in macrophages may be diminished or altered, leading to changes in the expression of pro-inflammatory cytokines and other immune response genes (28). This could be one of the molecular mechanisms underlying the impact of NLRP3 depletion on macrophages and its effect on cancer biology, as observed in this study.

Interestingly, here we also found that macrophage-depletion of NLRP3 significantly reduced cytotoxic T cells in the EMC tumor, likely due to the altered interaction between TAMs and lymphocytes (29), which is consistent with a previous study that demonstrated a role of macrophagic NLRP3 signaling in regulation of T-cell differentiation and a phenotypic shift between tumor-suppressing type 1 T helper cells and tumor-promoting type 2 T helper cells (30). Indeed, T cells play a critical role in the immune response to cancer. Moreover, CD4+ T cells can differentiate into various subpopulations with distinct functions, including Th17 cells and regulatory T cells (Tregs). Th17 cells promote inflammation and stimulate the immune system to attack cancer cells, while Tregs suppress immune responses and promote immune tolerance. The balance between Th17 and Treg cells in the tumor microenvironment can impact the outcome of cancer development and progression through their crosstalk with macrophages. Understanding the dynamics and modulation of these T-cell subpopulations and their interaction with macrophages is important for the development of immunotherapeutic strategies against cancer. A future study may address the crosstalk between macrophages and T cells through NLRP3 signaling in EMC to fully understand the role of oxidative stress in tumorigenesis and to develop new strategies for targeting oxidative stress in the treatment of EMC.

## Data availability statement

The original contributions presented in the study are included in the article/supplementary material. Further inquiries can be directed to the corresponding authors.

## Ethics statement

The animal study was reviewed and approved by Shanghai Jiao Tong University.

## Author contributions

XZ, YX, JW, ZX, TQ, and JC are responsible for data acquisition and analysis. XZ, YX, TQ, and JC are responsible for study conception and design, data acquisition and analysis. XZ wrote the manuscript. TQ and JC are responsible for funding and are the guarantee of the study. All authors contributed to the article and approved the submitted version.
